# Reproductive Flexibility: Genetic Variation, Genetic Costs and Long-Term Evolution in a Collembola

**DOI:** 10.1371/journal.pone.0003207

**Published:** 2008-09-15

**Authors:** Thomas Tully, Régis Ferrière

**Affiliations:** 1 Laboratoire Fonctionnement et Évolution des Systèmes Écologiques, CNRS UMR 7625, École Normale Supérieure, Paris, France; 2 Institut Universitaire de Formation des Maîtres, Université Paris 4, Paris, France; 3 Department of Ecology and Evolutionary Biology, University of Arizona, Tucson, Arizona, United States of America; University of Edinburgh, United Kingdom

## Abstract

In a variable yet predictable world, organisms may use environmental cues to make adaptive adjustments to their phenotype. Such phenotypic flexibility is expected commonly to evolve in life history traits, which are closely tied to Darwinian fitness. Yet adaptive life history flexibility remains poorly documented. Here we introduce the collembolan *Folsomia candida*, a soil-dweller, parthenogenetic (all-female) microarthropod, as a model organism to study the phenotypic expression, genetic variation, fitness consequences and long-term evolution of life history flexibility. We demonstrate that collembola have a remarkable adaptive ability for adjusting their reproductive phenotype: when transferred from harsh to good conditions (in terms of food ration and crowding), a mother can fine-tune the number and the size of her eggs from one clutch to the next. The comparative analysis of eleven clonal populations of worldwide origins reveals (i) genetic variation in mean egg size under both good and bad conditions; (ii) no genetic variation in egg size flexibility, consistent with convergent evolution to a common physiological limit; (iii) genetic variation of both mean reproductive investment and reproductive investment flexibility, associated with a reversal of the genetic correlation between egg size and clutch size between environmental conditions ; (iv) a negative genetic correlation between reproductive investment flexibility and adult lifespan. Phylogenetic reconstruction shows that two life history strategies, called HIFLEX and LOFLEX, evolved early in evolutionary history. HIFLEX includes six of our 11 clones, and is characterized by large mean egg size and reproductive investment, high reproductive investment flexibility, and low adult survival. LOFLEX (the other five clones) has small mean egg size and low reproductive investment, low reproductive investment flexibility, and high adult survival. The divergence of HIFLEX and LOFLEX could represent different adaptations to environments differing in mean quality and variability, or indicate that a genetic polymorphism of reproductive investment reaction norms has evolved under a physiological tradeoff between reproductive investment flexibility and adult lifespan.

## Introduction

All organisms experience environmental variation, and environmental variation is a fundamental ingredient of the evolution of organismal diversity. Life history attributes are, by definition, closely tied to Darwinian fitness and they occur in extraordinarily diverse combinations [1], [2]; therefore life history evolution should be particularly revealing about the relation between environmental variation and evolutionary change [1], [3].

How environmental variation influences the evolution of life history traits depends on the scale over which environmental conditions vary [2]–[5]. When environmental variation operates on large temporal and/or spatial scales compared to population persistence or dispersion, constant, genetically fixed traits are expected to evolve within populations, and variation to evolve between populations. When the temporal/spatial scale of environmental variation is commensurate to the organism's generation time or home range, the evolution of developmental plasticity is expected, whereby the individual's traits are fixed by the environmental conditions experienced during ontogeny.

When environmental variation occurs on even faster/shorter scales, an individual is likely to experience different environmental conditions during its lifetime. Fast/short-scale environmental variation can select for life history strategies that consist in genetically determined rules by which single individuals respond to environmental fluctuations. The strategy may be purely probabilistic, as with so-called bet-hedging strategies [6], [7], where the rule is reduced to expressing a certain trait (or trait value) with a genetically determined probability. When environmental variation has some degree of predictability, another type of adaptation is expected: ‘phenotypic flexibility’ (also called ‘flexible phenotypic plasticity’ [8], ‘reversible phenotypic plasticity’ [9], ‘facultative adjustment’ [10] and ‘context dependence’ [11], [12]), i.e. the rapid adjustment of labile phenotypic traits in response to fast/short scale variation in environmental conditions. One would expect life history flexibility to be a common adaptation to microenvironmental variability. Yet surprisingly little is known, both theoretically and empirically, about the occurrence and evolution of adaptive life history flexibility [13]–[15].

Phenotypic flexibility is expected to evolve in fitness traits, of which egg size has received much attention as a form of pre-natal maternal care enhancing the chance of offspring survival under adverse environmental conditions [2], [16]. Egg size along with clutch size and reproductive investment form a complex of functionally related traits [16], [17]. Our research program aims at investigating the phenotypic expression, genetic architecture, and long-term evolution of flexibility in this complex of reproductive traits, in response to rapid changes in food and social conditions. Arthropods have provided outstanding model systems for the study of life history evolution [16]. Here we introduce the collembola *Folsomia candida*, a widespread parthogenetic microarthropod, as a new model organism with several interesting features for the evolutionary analysis of life history traits in variable environments: an asexual reproductive system, a relatively short generation time, a high sensitivity to environmental conditions, including food availability, and the feasibility of non-invasive, semi-automated counting and measurements of individuals and eggs.

In this report, we address the following questions: (1) How flexible are egg size, clutch size, and maternal reproductive investment in response to sudden changes in dietary and social conditions? (2) Does the degree of flexibility differ between traits? (3) What fitness benefits do reproductive adjustments carry? (4) How much genetic variation is there in the mean and flexibility of reproductive traits? (5) What are the consequences of different amounts of genetic variation in the flexibility of different traits on the genetic correlations observed under different environmental conditions? (6) How did contemporary variation in reproductive flexibility evolve?

Our experimental investigation of reproductive flexibility uses the parthenogenetic (all-female) springtail *Folsomia candida* Willem (Collembola, Isotomidae) [18] as model organism (see Materials and Methods, section A). Springtails from eleven genetically distinct clones [19] were kept in harsh environmental conditions set by high density and low food ration. After about three months, individuals were isolated. Then they were fed *ad libitum* and their body size and reproductive behavior (egg size, clutch size, reproductive investment) were monitored for two weeks (see Materials and Methods, section B, Experiments 1 and 2, and section C). This experimental design enabled us to study how the reproductive traits covaried plastically in response to the environmental change between ‘bad’ and ‘good’ conditions. By comparing the eleven clones, we could also measure the genetic variability (heritability) of these traits and their flexibility (see Materials and Methods, sections C and D). A separate experiment was performed to assess the adaptive value of flexible adjustments, by measuring the relationship between egg size, offspring size and offspring survival under either bad or good conditions (see Materials and Methods, section B, Experiment 3, and section D). We looked for potential costs of flexibility by correlating a measure of reproductive flexibility with mortality rates among clones. Finally, we used the phylogeny of the clones to perform a comparative analysis of their flexible traits, in order to gain insight into the origin and diversification of reproductive flexibility (see Materials and Methods, section D).

## Results

### Reproductive traits are flexible

**Figure 1:**
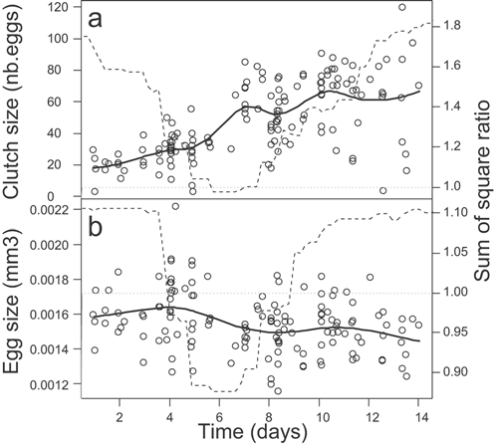
Reproductive adjustments after release of crowding and dietary restriction: (a) egg size (mean per clutch), (b) individual clutch size. Solid line: smooth spline function fitted to data. The effect of time on egg size and clutch size was analyzed by contrasting two linear models: *Egg size (or Clutch size) = Body length+Clone+Time* (model 1), and *Egg size (or Clutch size) = Body length+Clone+Period* (model 2). Model 2 involved two consecutive periods; by varying the limit between the two periods, we could examine whether specific parameterization of model 2 made discrete time (period effect) a better model than continuous time. The two models were compared by means of the ratio of the residual sum of square (dashed line). For both egg size and clutch size, model 2 became superior to model 1 when the period limit was close to 6 days (ratio<1). Note that the plotted values of clutch size and egg size are values corrected for female body length (i.e. the measurements are standardized for a 1.6 mm long individual).

A marked decrease in egg size associated with increasing clutch size occurs 6 days after the release of crowding and dietary restriction (Figure 1a, b)— a time lag that exactly equals the minimal inter-clutch interval (mean inter-clutch interval = 6.7 days, 95% confidence interval = [5.9; 8.9], n = 51). Clutches laid during the first period (P1, day 1 to 6) come from a reproductive cycle that began in the crowded-dietary restricted environment. Clutches laid during the second period (P2, from day 7 onward) are on average composed of smaller (−7.5%, χ^2^_1_ = 30.7, P<0.001) but more eggs (+231%, χ^2^_1_ = 89.8, P<0.001) than in P1 (Figure 1).

In the control experiment (see Materials and Methods, section B, experiment 2), when controlling for clone, food ration and body size, we found no effect of maternal age on egg size (χ^2^_1_ = 0.39, P = 0.53) and a negative effect of maternal age on clutch size (−0.2 egg/day, χ^2^_1_ = 82, P<0.001) which is much smaller than, and opposite to the treatment effect evidenced in the main experiment. Thus, the period effect is likely to be due to the sudden change in environmental conditions rather than to a confounded effect of maternal age.

### Reproductive adjustments confer fitness benefits

In order to probe the adaptive significance of reproductive flexibility, we assessed the effect of environmental conditions (crowded and dietarily restricted conditions versus isolation and full feeding) on maternal reproductive investment and the relation between egg size and juvenile quality (see Materials and Methods, section B, experiment 3). The Winkler-Wallin optimality model [2], [20] makes a key prediction from the adaptive hypothesis: under poor environmental conditions [16], [21], low maternal reproductive investment is expected while bigger eggs associated with greater nutritional provisions should result in larger larvae that survive better.

**Figure 2:**
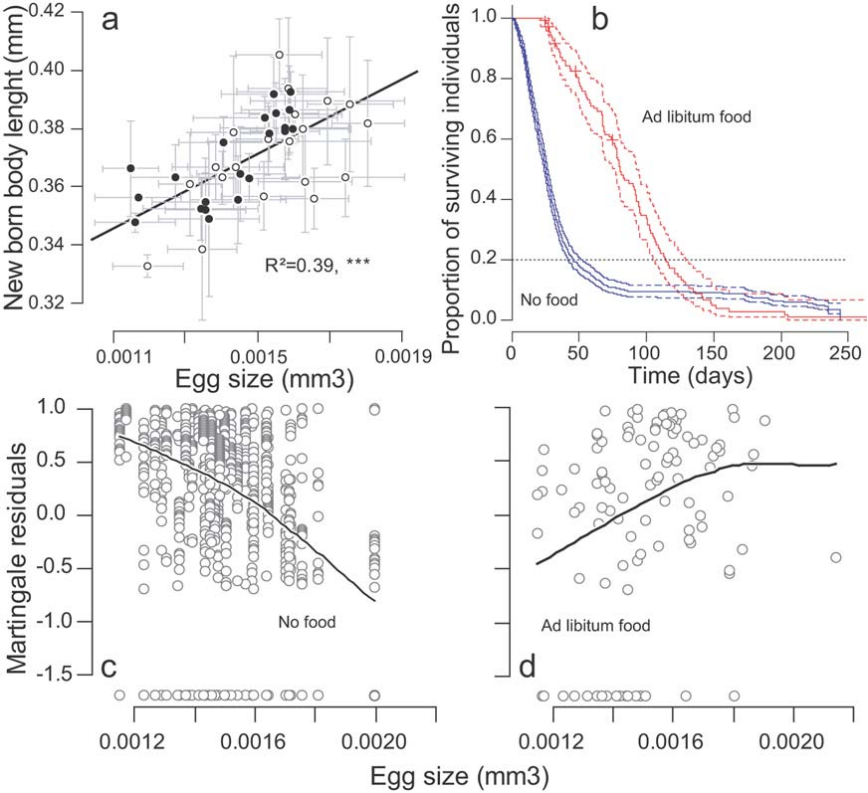
Egg size, offspring size, and offspring survival in the two environments (Experiment 3). (a) Correlation between egg size and newborn body length. Egg size and newborn body length size (mean per clutch +/− SE) are positively correlated. Measurements from 20 clutches laid during the first period (open circles) and 21 clutches laid during the second period (closed circles). (b) Survival curves (and 95% confidence intervals) in the no food (and high density) and *ad libitum* (and low density) food treatments. In the survival analysis we used the death events that occurred over the dotted horizontal (80% limit, see Materials and Methods, section D). Some individuals in the no food treatment survived very long probably because they could scavenge on dead bodies. (c) and (d) Association between egg size (mean per clutch) and offspring survival, depending on food availability. Martingale residuals are computed from Cox proportional hazard models *not* including egg size as a covariable (see methods for details). Non randomness in the residuals is evidenced by a local polynomial regression fit (curves, computed using *scatter.smooth* function in software R 2.1). (c) In the no food environment, residuals decrease with egg size: neonate issued from large eggs were more represented among old survivors, whereas those that hatched from small eggs had a higher mortality rate. (d) With food *ad libitum*, mortality rate tend to increase (not significantly) with egg size.

The prediction is upheld in *F. candida*. Reproductive investment is uniformly low among clones in period P1, and rises significantly in P2 (clutch size multiplied by 2.5, χ^2^_1_ = 9.67, P = 0.0018). Body length measured within 20 h after birth on 210 neonates from 41 clutches was positively correlated to the mean volume of the eggs from which they hatched (cor = 0.64, 95%CI = [0.41; 0.79], t_39_ = 5.2, P<0.001, Figure 2a). Offspring survival was affected by dietary and crowding conditions: the mortality rate was multiplied by 12 under high density and starvation (95%CI = [6.2; 23.4], |z| = 7.3, P<0.001, Figure 2b). Moreover, survival was affected by an interaction between dietary/crowding conditions and mean egg size (|z| = 2.8, P = 0.005, Figure 2c, d). During the first month of life, under high density and food deprivation, clutches containing larger eggs produced individuals surviving longer than clutches with smaller eggs (|z| = 3.94, P<0.001, Figure 2c): a 10% increase in egg volume decreased the mortality rate by 31% (95%CI = [17%; 43%]). In contrast, under low density and full feeding variation in egg size did not affect survival (|z| = 1.02, P = 0.31, Figure 2d).

### How do reproductive adjustments vary among individuals?

**Figure 3:**
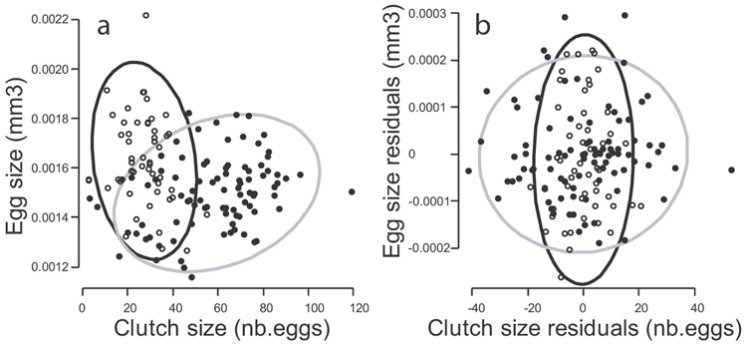
Phenotypic correlation structure of egg size and clutch size. Open circles: data from period P1, closed circles: period P2. The 90% concentration ellipses are indicated for both periods. For each measurement of clutch and egg size, maternal body length is taken into account and standardized to 1.6 mm. (a) Global phenotypic correlations between egg size and clutch size. (b) Within-clones residuals correlations between egg size and clutch.

In order to analyze the structure of variation of reproductive adjustments among individuals, we begin with an examination of within-environment patterns. Egg size is related to clutch size (controlled for mother's body length) differently among periods (χ^2^_1_ = 6.14, P = 0.013, Figure 3a): in P1, egg size shows a negative yet non-significant correlation with clutch size (cor = −0.19, 95%CI = [−0.44; 0.08], t_52_ = −1.4, P = 0.16), whereas in P2, females that produce larger clutches also lay bigger eggs (cor = 0.27, 95%CI = [0.06; 0.45], t_86_ = 2.61, P = 0.01). These results contrast with the classic assumption of a negative correlation (tradeoff) between egg size and clutch size. In fact, many studies have demonstrated a phenotypic tradeoff between offspring size and number [16], but few of them have controlled for underlying genetic differences between individuals [22]. How much does genetic variation contribute to variation in egg size, clutch size, and reproductive investment within each period? When taking within-period genetic variation into account, no physiological tradeoff between egg size and clutch size could be detected: within-clones residuals for egg size and clutch size are not correlated, neither in P1 (t_52_ = 0.005, P = 0.99) nor in P2 (t_86_ = 0.33, P = 0.74, Figure 3b).

**Figure 4:**
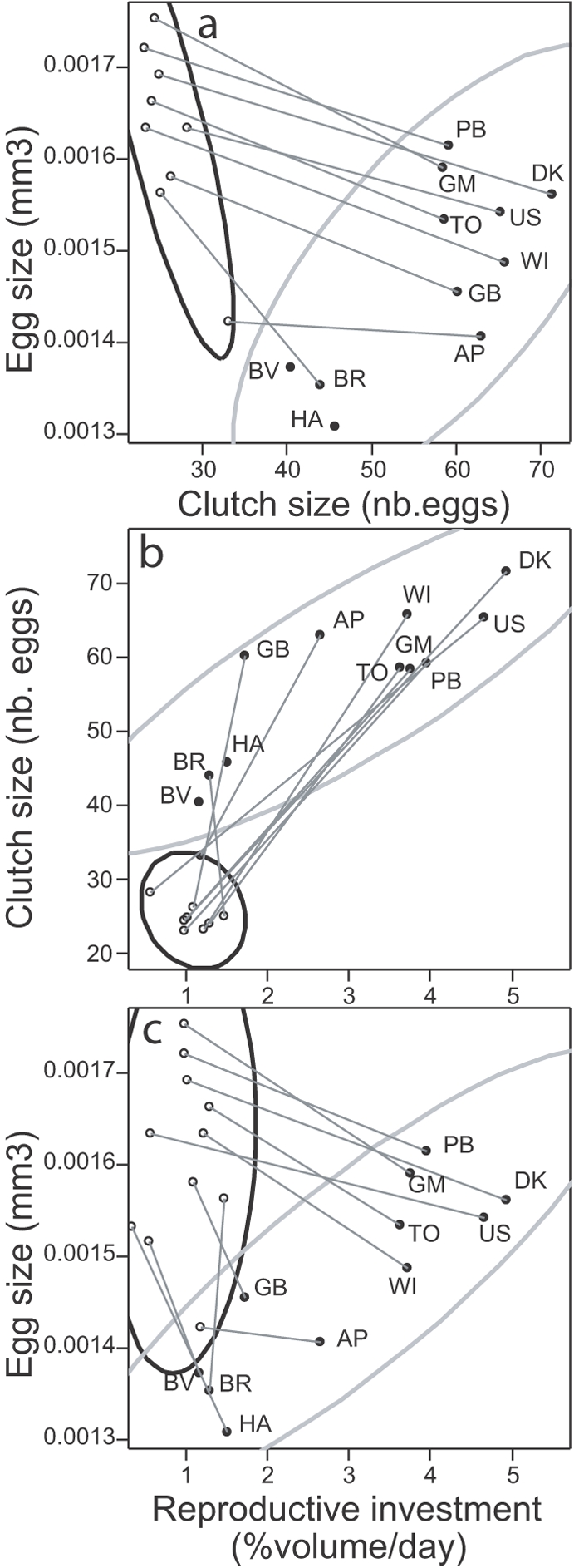
Genetic correlations between egg size, clutch size and reproductive investment. Bivariate reaction norms (grey lines) and 90% concentration ellipses for period P1 (open circles) and P2 (filled circles). (a) Egg size and clutch size. (b) Clutch size and reproductive investment. (c) Egg size and reproductive investment. Only data from period P2 have been plotted for clones BV and HA because these clones laid too few eggs in period P1. The measurements are standardized for a 1.6 mm long female.

Despite a high level of intra-clutch egg volume variation that account for 50% of total variance, egg volume expressed in each environment was found to be highly heritable (H^2^ = 25%, 95%CI = [21], [29], χ^2^_1_ = 49.8, P<0.001). During P1 genetic variation had no effect on clutch size (χ^2^_1_ = 0.79, P = 0.37, Figure 4a) or reproductive investment (χ^2^_1_ = 0.26, P = 0.88, Figure 4b) whereas in P2 both traits were found to be heritable (clutch size: H^2^ = 42%, 95%CI = [14; 67], χ^2^_1_ = 10.6, P = 0.001; reproductive investment: H^2^ = 48%, 95%CI = [30; 64], χ^2^_1_ = 34.6, P<0.001). Egg size and clutch size are genetically correlated within each period, and, remarkably, these genetic correlations are reversed between periods: from negative in P1 to positive in P2 (Figure 4a; in P1: cor = −0.81 [−0.96; −0.31], t_7_ = −3.64, P = 0.008; in P2: cor = +0.70 [0.18; 0.92], t_10_ = 2.96, P = 0.016). Genetic correlations between reproductive investment and egg size or clutch size are nonsignificant in P1, but are strongly positive in P2 (Figure 4b, c. Egg size and reproductive investment in P1: cor = +0.14 [−0.50; 0.68], t_9_ = 0.44, P = 0.67; in P2: cor = +0.87 [0.56; 0.96], t_9_ = 5.22, P<0.001. Clutch size and reproductive investment in P1: cor = −0.18 [−0.75; 0.55], t_7_ = −0.48, P = 0.64; in P2: cor = +0.84 [0.49; 0.96], t_9_ = 4.71, P = 0.001).

### Genetic variation in reproductive flexibility

**Figure 5:**
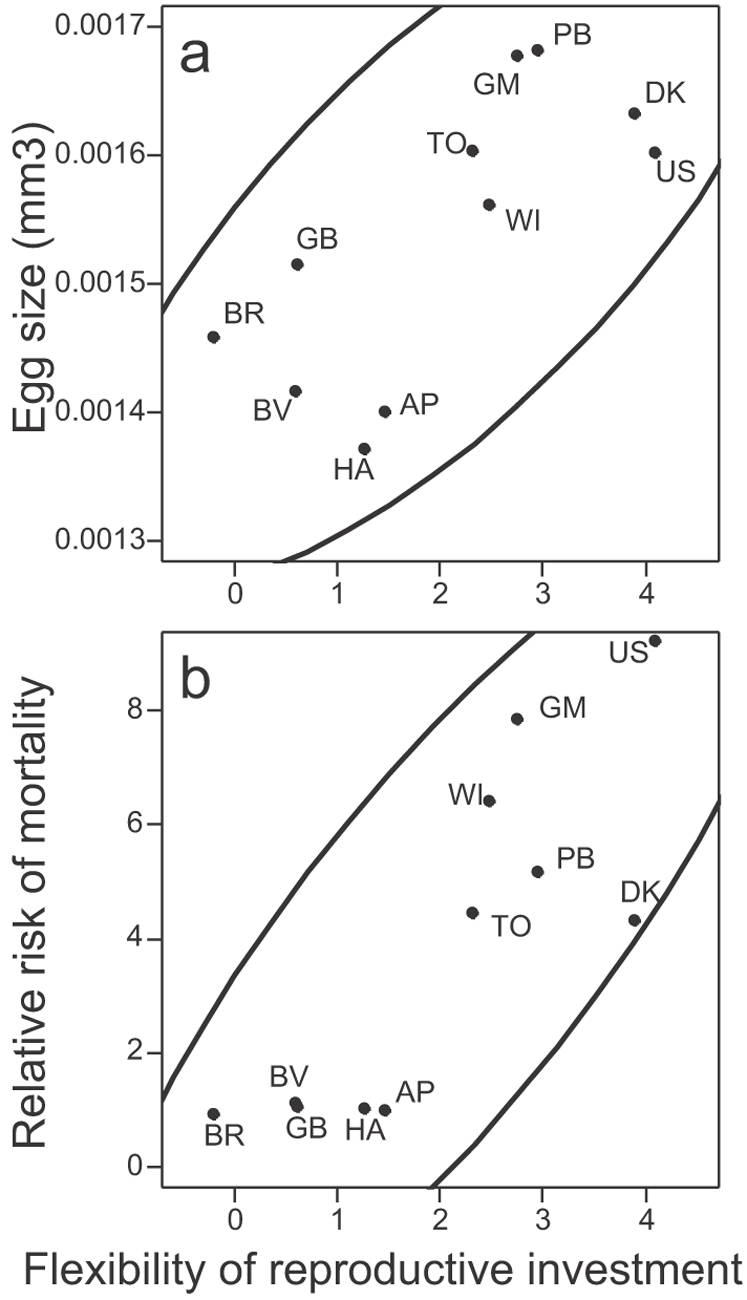
Genetic correlations between flexibility of reproductive investment and (a) egg size, (b) adult mortality. 90% concentration ellipses are indicated. Genetic values of relative risk of mortality (clone AP is taken as a reference with a relative risk of one) come from an independent experiment where the longevity of 20 individuals per clone was measured and analyzed through a Cox proportional hazard model. Mortality risk differs among clones (χ^2^_1_ = 109, P<0.001). For each measurement of egg size and flexibility of reproductive investment, maternal body length is taken into account and standardized to 1.6 mm.

The great variation of genetic correlations between egg size, clutch size and reproductive investment is the consequence of flexibility in these traits, and an amount of genetic variation in flexibility that differs among traits [23]. There is no genetic variation in egg size flexibility (χ ^2^_1_ = 0.01, P = 0.92) whereas there is strong genetic variation in the flexibility of reproductive investment (H^2^ = 34.5%, 95%CI = [18.2; 49.8], χ ^2^_1_ = 23.0, P<0.001). In effect, the degree of flexibility in reproductive investment varies from no increase in clone BR to an 8-fold increase in clone US (Figure 4b, c). Thus, whereas all genotypes show a similar response in egg size to environmental change, the degree to which clutch size is affected is not simply determined by a physiological trade-off with egg size—it also integrates the flexibility of maternal investment in reproduction. In genotypes producing consistently (i.e. on average across periods) bigger eggs, reproductive investment is more flexible (Figure 5a; correlation of genetic values of mean egg size over both periods with genetic values of reproductive investment flexibility: cor = +0.74 [0.26; 0.93], t_9_ = 3.36, P = 0.008), and disproportionately larger clutches are produced under favourable conditions, as permitted by a larger increase of reproductive investment.

This suggests that resource acquisition strategies may differ among clones [17], [24]. According to this interpretation, under crowded conditions and food deprivation, genetic variation in resource acquisition is weakly expressed and only genetic variation in resource allocation is detected, leading to the negative genetic correlation between clutch size and egg size (Figure 4a). In contrast, under isolated conditions and full feeding, genetic variation in resource acquisition is fully expressed, thus masking genetic variation in resource allocation and leading to positive correlations between egg size, clutch size and reproductive investment (Figure 4).

The maintenance of genetic variation in reproductive flexibility could thus be explained by the tradeoff that life history theory predicts between resource acquisition strategies and survival [25], [26]. Specifically, the tradeoff hypothesis implies that the resource acquisition strategy underlying high flexibility in reproductive investment should suffer the genetic cost of higher adult mortality. This hypothesis is supported by our data: the adult risk of mortality is higher in clones that cumulate the benefits of larger egg size in both periods (correlation between genetic values of mean egg size and mortality risk (relative to clone AP): cor = +0.79 [0.37; 0.94], t_9_ = 3.9, P = 0.003) and high flexibility in reproductive investment (correlation between genetic values of reproductive investment flexibility and mortality risk: cor = +0.84 [0.48; 0.96], t_9_ = 4.6, P = 0.001, Figure 5b).

### Genetic costs and long-term evolution of reproductive flexibility

**Figure 6:**
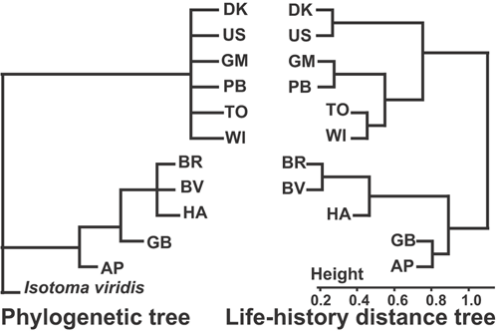
Phylogeny and life history evolution. The phylogeny (left) is a strict consensus cladogram with proportional branch lengths obtained by analyzing two types of molecular characters (RAPD markers and rRNA sequences [19]). The collembola *Isotoma viridis* Bourlet was used as an outgroup. The topology of the upper clade is unresolved due to contradictory signals—not because of lack of genetic variation. The life history distance tree (right) was derived from a hierarchical cluster analysis performed on the genetic values of egg size and reproductive investment expressed in P2 (cf. Figure 4c and methods). The two trees are highly congruent (comparison of the two associated distance matrices, 1000 permutations, Friedman's χ^2^_1_ = 96, P<0.001).

A hierarchical cluster analysis (see Materials and Methods, Section D) made on the genetic values of egg size and reproductive investment highlights the existence of two genetically distinct reproductive strategies (Figure 4c, Figure 6): a high-flexibility strategy, HIFLEX, characterized by larger egg size and highly flexible reproductive investment (clones DK, US, GM, PB, TO, WI), and a low-flexibility strategy, LOFLEX, which produces small eggs in both periods and barely increases its reproductive investment in response to the environmental amelioration (clones BR, BV, HA, GB, AP).

This genetic clustering into HIFLEX and LOFLEX strategies shows remarkable congruence with the clones' phylogeny (Figure 6). The two strategies arose once along with the early divergence of two major branches of the evolutionary tree, and the distribution of genetic trait values measured in P2 (Figure 4c) almost perfectly matches the subsequent branching structure of the tree.

## Discussion

Phenotypic plasticity can exist in various guises, which are encapsulated theoretically by the concept of ‘reaction norm’—the potential phenotypic response to different environments (see [8] and [27] for reviews). Reaction norms can be either *inflexible*, in which a characteristic once determined is never changed later in the organism's life, or they can be *flexible*, in which a characteristic can be altered more than once in the development of the same individual. To date, life history theory has focused on life history traits, such as growth rate or age at maturity, whose phenotypic variation is described by inflexible reaction norms [8], [9], [28]. In animals, the evolutionary analysis of life history flexibility has been limited chiefly to maternal adjustment of sex ratio [10] and sex allocation [28], and to the context-dependent expression of sexual traits [11], [29] and offspring dispersal [30], [31].

Here we have shown that collembola are capable of remarkably fast and large adjustments of their reproductive traits (reproductive investment, clutch size, egg size) in response to sudden environmental change in food and density conditions. We documented the phenotypic expression, genetic variation, and long-term evolution of reproductive flexibility by means of a comparative analysis of eleven clones from different origins worldwide. Our results (i) provide evidence for the adaptiveness of reproductive flexibility, (ii) reveal that genetic variation in flexibility differs between traits (which has consequences for the observed genetic correlations between traits in the different environments experienced by individuals during their lifetime), and (iii) suggest the importance of resource acquisition tradeoffs to understand the origin, maintenance and evolution of genetic variation in the flexibility of resource allocation traits.

Hereafter we discuss our results mostly in the light of recently published analyses of adaptive plasticity of egg size, which in many cases might pertain to the flexible kind documented in our study system. Thus, the growing understanding of the evolution of egg size plasticity provides a useful background for interpreting and discussing our results.

### Adaptive flexibility of egg size

As for any biological trait, the adaptive hypothesis implies heritable variation, and differential costs and benefits. In *Folsomia candida*, egg size strongly correlates with offspring size, and offspring size has a marked, positive effect on juvenile survival under poor food conditions, thus providing evidence for a fitness benefit from egg size adjustment. Although there is no genetic difference between clones in egg size plasticity, the high heritability of egg size in both periods supports the genetic basis of the egg size reaction norm. Thus, our analysis add to a relatively short list of experimental studies that have demonstrated cross-generational adaptive plasticity via maternal manipulation of offspring size, mainly in invertebrate model systems—*Daphnia* [32], the seed beetle *Stator limbatus* [33], the tropical butterfly *Bicyclus anynana* [34], and the bryozoan *Bugula neritina* [31]—and in the Trinidadian guppy *Poecilia reticulata* [25], [35]. The reversible plasticity, i.e. flexibility, of egg size has been documented in the Ural owl *Aegolius funereus*, a long-lived bird that preys on highly fluctuating populations of voles; pedigree analysis and strong correlative evidence show that egg size is heritable and adjusted in response to variation in prey density, and supplementary experiments suggest that these adjustments do confer fitness benefits [15]. In the common lizard *Lacerta vivipara*, life history flexibility manifests itself in response to multiple cues, but its putative fitness benefits remain elusive [36], [37].

Because of the effect of egg size on individual fitness, egg size has long been viewed as a relatively canalized trait in animals (see references in [38], [39])—an assumption that has been revisited in the light of growing evidence for genetic variation in egg size [38]. In beetles [40] and guppies [35], there is genetic variation for egg size mean *and* plasticity. A selection experiment in beetles found that selection for increased egg size resulted in increased egg size plasticity, but only in one particular environment [41], whereas in guppies, increased offspring size plasticity was associated with decreased offspring size [35]. In our collembola, lack of genetic variation in egg size flexibility may indicate canalization or convergent evolution. In either case, our results suggest that the evolution of mean egg size can be relatively decoupled from the evolution of egg size flexibility, due to constraints (e.g. egg size flexibility hit its physiological limit, as discussed below) or because the determination of mean egg size and the regulation of egg size flexibility involve different genes or genetic pathways [41], [42].

### Environmental variation and genetic correlations

Within periods, egg size genetically correlates with clutch size and reproductive investment. These genetic correlations show a striking reversal between periods, from strong negative in the bad period to strong positive in the good period. This finding adds to growing empirical evidence that genetic correlations can shift, even switch sign, across environments [22], [43]; our results are distinctive as they demonstrate reversals of genetic correlations *within the individual lifetime*.

The bad period is characterized by uniformly low reproductive investment among clones. This is consistent with the hypothesis of unfavorable conditions decreasing heritability as a consequence of selection favoring alleles (in loci promoting resource allocation to reproduction) that are not expressed in periods of food shortage 44, 45. Poor environmental conditions generate strong viability selection on egg size. Thus, the negative genetic correlation between egg size and clutch size in the bad period is consistent with the classic hypothesis that egg size and clutch size are optimized by selection, with harsher environmental conditions favoring larger eggs in smaller clutches [21]. The intriguing result, however, is that the physiological tradeoff expected to constrain the optimization process [2], [21], [46] could not be detected. When controlling for period, maternal size and genotype, no relation exists between egg size and clutch size in either environment (Figure 3b). This puzzling result warrants further investigation.

In the good period, genetic variation is expressed in all three reproductive traits: egg size, clutch size, and reproductive investment. The evolution of reproductive investment has long been regarded as decoupled from the evolution of clutch size and egg size [21], but recent empirical studies have cast doubt on this fundamental assumption [22], [34], [47]. Yet even for constant environments, surprisingly little theory is available to predict the outcome of the joint evolution of egg size, clutch size, and reproductive investment. The Winkler-Wallin model [20] remains the chief theory for the joint evolution of all three traits; it predicts that better environmental conditions should select for larger reproductive investment, smaller eggs, and larger clutches—disproportionately so as a consequence of larger reproductive investment. The genetic correlation found in the good period conforms only partly to that prediction: larger reproductive investment is associated with larger clutches but also larger, rather than smaller eggs; and a larger offspring size is expected to evolve under harsher, not milder environments. How can we resolve these discrepancies? Variation in the expression of flexible traits across environments cannot be fully understood without considering the evolution of flexibility itself [5], [24], [48].

### Limits and costs of reproductive flexibility

In contrast with egg size flexibility, the flexibility of reproductive investment shows substantial genetic variation in *Folsomia candida*. Reproductive investment flexibility is genetically and positively correlated with mean reproductive investment and mean egg size. A harsher environment that selects for larger mean egg size may also promote a greater ability to adjust reproductive investment in response to more intense or more frequent environmental fluctuations, e.g. a higher rate of transition from good to bad conditions [9], [45]. Larger mean reproductive investment may then evolve simply as a consequence of a steeper reaction norm [49].

How limited or constrained would the evolution of reproductive flexibility be? Egg size flexibility does not seem limited by a response time lag [48]: reproductive traits can be adjusted even once the individual's reproductive cycle has started, which suggests that more energy can be channelled into reproduction as soon as new resources become available (see Figure 1b: those clutches laid during P1 follow a trend for larger size as the laying date advances: +2.9 eggs/day, χ ^2^_1_ = 8.9, P = 0.003). But the common pattern of egg size adjustments across clones might reveal the ‘phenotypic range’ limit of plasticity [48]. Thus, the lack of genetic variation in egg size flexibility would be consistent with a common physiological limit hit by the evolution of egg size flexibility in all populations. As a consequence, the minimum egg size expressed in good environments would be consistently greater in populations evolving higher mean egg size. Alternatively (yet non exclusively), larger size at birth might evolve as a correlated response to selection for larger reproductive investment [35].

Higher adult mortality has been hypothesized as a potential genetic cost for increasing reproductive plasticity [26]; our finding of a strong positive genetic correlation between reproductive investment flexibility and adult mortality upholds this prediction. Consistently with our experimental results, correlational data in Ural owls also show that the most reproductively flexible individuals have shorter reproductive lifespan [15]. In contrast, the experimental analysis of the mean and plasticity of survival, growth, and reproductive effort in the Pacific oyster *Crassostrea gigas* raised under different food conditions, revealed substantial genetic variation in reproductive effort plasticity and in mean survival; but the degree of plasticity in reproductive effort and mean survival covaried positively [24] —a pattern explained by hypothesizing that reproductive effort plasticity trades off with sensitivity to random factors of mortality [24]. We also expect that in more variable environments, higher adult mortality and higher reproductive investment co-evolve along a basic reproduction/survival trade-off [50], [51]. In collembola, the positive genetic correlation between mean egg size and mean reproductive investment, and the negative covariation of mean egg size or mean reproductive investment with adult survival are also compatible with that prediction. The complexity of the picture exemplifies how challenging the measure of genetic costs of phenotypic plasticity remains [52]–[54].

### Evolutionary scenarios

**Figure 7:**
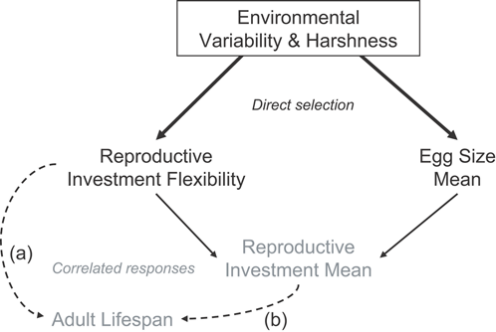
Adaptive scenarios for the evolution of reproductive investment flexibility. Harsher and more variable environments select for higher mean egg size and higher flexibility in reproductive investment. Larger mean reproductive investment and shorter adult life span evolve as correlated responses (gray). Scenario (a) emphasizes a tradeoff (dotted arrow) between adult lifespan and reproductive investment flexibility. Alternatively, scenario (b) emphasizes a tradeoff between adult lifespan and reproductive investment mean.

Genetic correlations obtained from clones of vastly different origins (when it is known ) may reveal patterns of adaptations to a range of selective environments experienced by each of the original populations. We know from laboratory experiments that collembola population dynamics can respond dramatically to changes in patterns of environmental variation and autocorrelation [55] and trophic interactions [56]. Thus, changes in environmental harshness and variability are likely to affect the outcome of competition between genetic variants. In this context, our interpretations of genetic correlations yield two main adaptive scenarios (Figure 7), in which different life history adaptations evolve in response to different degrees of environmental harshness and variability. Resolving these alternate scenarios requires that we learn more about the ecology and population genetics of natural collembola populations. We also need to elucidate the physiological basis of resource allocation between life history traits and their flexibility [41], [57]. Indeed, genetic correlations that reflect different adaptations among populations provide little insight into the structure of physiological tradeoffs that prevail in each population. To this end, the expression of different traits values across environments by the same individuals during their lifetime may present new and fruitful opportunities [13].

The interpretation of genetic differentiation as a response to different selective environments is tantalizing but remains hypothetical. A contrasting view would assume that the genetic variation documented here actually reflects the genetic polymorphism of natural populations. In this case, the single origin and evolutionary divergence of HIFLEX and LOFLEX could be interpreted as the result of disruptive selection operating on a single (i.e. common to all populations) tradeoff between adult survival and reproductive investment mean or flexibility [58]. The breakdown of genetic correlations at smaller phylogenetic scale might indicate that the fitness landscape over which phenotypes evolve becomes flatter away from the original branching phenotype – an assumption that is consistent with theory [58], the empirical analysis of egg size flexibility in Ural owls [15], and the hypothesis of nonlinear selection to explain the breakdown of genetic correlations in laboratory evolution of *Drosophila* [59]. A similar effect – dependence upon phylogenetic scale of the tradeoff underlying variation in reaction norms – was suggested by data on thermal reaction norms of body growth in fish [60].

### Perspectives

The evolution of life history flexibility, i.e. the adaptive, context-dependent adjustment of fitness traits by individuals during their lifetime, raises exciting challenges at the crossroads of genetics, physiology, ecology and evolution. While future work on the collembola system may afford further insights into how life history traits evolve as reaction norms, there is an urgent need to develop general models and theory that will form the conceptual framework of empirical studies. Simple theoretical models of the evolution of life history traits are of limited value in heterogeneous environments in which complexes of traits covary and thus co-evolve, and the complex of traits that coevolve varies with environmental conditions [22]. There are still very few general models of the evolution of reversible plasticity [9], [49], and to our knowledge, none that involves the population physiological structure needed to address the evolution of flexibility in life history traits.

The development of an evolutionary theory for life history reaction norms will be useful to address the multidimensionality of environmental and physiological cues [61]–[64], to dissect the physiological and genetic architecture of flexibility in complexes of functionally related traits [13], [57], [65], and to investigate the reciprocal influence of phenotypic flexibility and evolutionary dynamics [66]–[71]. Mirroring research perspectives on developmental plasticity [72], one of the next frontiers will be to disentangle the web of ecological and evolutionary feedbacks between life history flexibility and the community and ecosystem contexts of population adaptation.

## Materials and Methods

### A. *Folsomia candida* as a model organism

*Folsomia candida* Willem 1912 (Collembola, Isotomidae) is a widespread parthenogenetic springtail [18] that is typically found in leaf litter, in caves [73], [74] and also in anthropic environments such as the dirt of plant pots [75]. Its natural density is known to vary greatly [76]. Individuals mature within two weeks and lay a clutch about once a week [77], [78]. Clutch size varies from less than ten eggs to more than 100; body length [79] and ration [75], [80], [81] are major influences of egg production.

Clonal populations issued from one single female for each strain are maintained in our laboratory. All populations and single individuals monitored during the experiments were maintained in standard containers made of a polyethylene vial (diameter 52 mm, height 65 mm) filled with a 30 mm layer of plaster of Paris mixed with 600 µL of Pebeo® graphic Chinese ink to increase visual detectability of individuals and eggs against their background. The surface of the plaster was sandpapered and covered with a thin layer of a mixture of clay, Chinese ink and charcoal in order to fill up all tiny holes in the plaster that springtails could have used to lay eggs. All direct manipulations were done by using a pooter (for individuals) and a thin moisturized brush (for eggs).

Food is provided in the form of small pellets of a mixture of dried yeast and agar in standardized concentration and volume (5000 µL water+80 mg agar+800 mg dried yeast, to produce pellets of 2 µL). All our stock cultures are provided with the same amount of food. Stock cultures and experimental populations are kept in incubators at 21±0.5°C, with a 12 h∶12 h light∶dark cycle and constant humidity (∼100%).

### B. Experimental design

We used eleven clones of *Folsomia candida* characterized by molecular markers [19] – nine from Europe (clones AP, BR, BV DK, GB, GM, HA, PB, TO) and two from North America (US, WI).

#### Experiment 1: Transfer experiment to measure reproductive flexibility

This experiment aimed at measuring the response of reproductive investment (egg size and clutch size, see section C for methodological details) to transfer from crowded and dietarily restricted conditions to isolation and full feeding. For each clone, four replicates of high density populations (ca. ∼40–50 ind./cm^2^) were provided with low food ration (∼1 µg dried yeast/ind/week) during three months. To mimic environmental amelioration, ten adult females of each clone were then isolated and fully fed (food pellets provided ad libitum). To reduce the influence of uncontrolled factors, we sampled young adults of similar size (size homogeneity between clones: F_10–99_ = 1.52, P = 0.14; mean body length = 1.47 mm, SE = 0.021).

#### Experiment 2: Control for age effect on egg size and clutch size

Because there is no monitoring the reproductive characteristics of single individuals in high density populations, a simple control experiment was not feasible. Therefore, to test for any confounding effect of age with environmental change, we performed a complementary experiment by measuring egg size and clutch size produced by 20 isolated individuals raised at two contrasted rations (low food and *ad libitum* food), for each clone and over four months. In the low food treatment, a food pellet was available one day per week whereas in the high food treatment, food was provided *ad libitum* seven days per week. These females were of the same age as those of the reported experiment (younger than four months).

#### Experiment 3: The effect of egg size on offspring survival in ‘good’ and ‘bad’ environments

The relationship between egg size and neonate size was documented by measuring body size in 210 neonates from 41 clutches within 20 h after hatching. The relation between egg size, juvenile size, and juvenile quality was assessed by measuring the survival of neonates in two contrasting environments: in the ‘bad’ environment, no food was provided to a cohort of ∼20 individuals; in the ‘good’ environment, food was provided *ad libitum* to isolated individuals. For each clone, the ‘bad’ environment treatment was carried out by isolating ca. 20 developed eggs obtained from four clutches laid by four females in the second week of the main experiment. For the clones GB and BR, only three clutches could be used, and only one for clone BV. The mortality curve of 811 neonates coming from these 39 clutches was estimated by monitoring the number of collembola still alive at regular time intervals. Each container was inspected twice a day until all the eggs had hatched, then every other day during one month.

In the ‘good’ environment treatment, 10 neonates issued from at least four different clutches were isolated for each clone immediately after birth and transferred to fresh rearing boxes. Unlimited food was provided to these 110 individuals by providing and regularly replacing food pellets (these individuals were also used in the *ad libitum* food treatment of the control experiment). The mortality curve was established by checking the boxes every day during three weeks, and every two to four days during the following three months. From month 4 to month 8 the boxes were inspected weekly.

Because we were unable to assign an individual egg size to each neonate, only the mean egg size of the corresponding clutch could be analyzed as a factor of juvenile body length or survival; intra-clutch egg size variation was not taken into account.

### C. Measurements and data collection

In the main experiment, rearing boxes were visually inspected twice a day (morning and evening) for clutches. When a new clutch was found, fecundity was measured by counting the eggs. Each clutch was then photographed with a digital camera (Nikon ® Coolpix 990) connected to an Olympus ® SZX12 stereomicroscope, after carefully spreading the eggs with a thin brush to facilitate egg contour detection through image analysis. Pictures were taken and egg size measurements (mean diameter and surface) were performed soon after the clutch had been laid (within 24 hours) to take advantage of the spherical shape of eggs (they become ovoid after the chorion tears, i.e. ∼after 3 days [75]. Egg size measurements were then converted into egg volume under the assumption of spherical shape. Digital pictures and image processing were also used to measure the body length of all females (from the front of the head to the rear of the abdomen) at the start and at the end of the first experiment and every week during the second experiment (control). We used the same method to measure the body length of new born individuals. Most females grew up during the experiments. We therefore estimated the body length of a female at each time she laid eggs by considering a linear body length growth trajectory during the intervals between two body length measurements.

We used the ImageJ software for image analysis [82]. The repeatability of egg size measurement was assessed in an independent experiment, by measuring 67 eggs issued from four clutches, each of which was shot four times yielding a total of 268 measurements. Likewise, 400 measurements of body length were obtained from ten pictures of eight adults, analyzed five times. Repeatability is defined as the proportion of variance associated with differences between individuals [83]. Repeatability scored very high for both egg size (79%), and body length (96%).

Overall, 93 of the 110 sampled females of the main transfer experiment laid at least one clutch; 51 laid two clutches. Of the 6627 eggs laid in the 144 clutches, 3377 were measured. Each clutch was assigned a maternal body length by assuming linear growth of the mother during the experiment. Maternal volume was estimated under a cylindrical shape approximation, by using body length and the relationship between body length and abdomen width estimated from an independent dataset from a preliminary experiment (abdomen width (mm) = 0.272*body length (mm) – 0.0536, R^2^ = 0.87, based on body size measurements made on 68 individuals ranging from 1.0 mm to 2.0 mm).

Reproductive investment was defined for each individual as the total volume of eggs produced during each period divided by the duration of the period and by the mean volume of the female during that period (%volume.day^−1^). The total volume of eggs was measured for each female by the sum over clutches of clutch volume, the latter being estimated by the product of egg number by mean egg volume. Our analysis of reproductive investment thus takes into account females' reproduction schedule and females that did not reproduce during one or both periods.

### D. Statistical analysis

#### Broad-sense heritabilities of reproductive traits' mean and flexibility

*Egg size*, *clutch size* and *reproductive investment* were analyzed by using hierarchical mixed linear models (*lme* function of *nlme* package, R 2.1) with *clone*, *mother* (for egg size, clutch size and reproductive investment) and *clutch* nested within *mother* (for egg size) as random effects [84]. For clonal organisms, the relevant measure of genetic variance is the broad-sense heritability defined as the ratio of the among-clone component of variance to the total phenotypic variance: H^2^ = σ^2^_G_/σ^2^_T_ [85]. Broad-sense heritabilities of the traits and of their flexibility, defined as the proportion of genetic to expected phenotypic variance for controlled body size, were calculated by using models with *clone* – for heritability of the mean trait – and interaction between *clone* and environment (*period*) – for heritability of the trait's flexibility – treated as random effects, and by comparing the variance component of these effects to the total variance. In the models used for computing heritabilities, variables of interest (*egg size*, *clutch size* and *reproductive investment*) are corrected for *maternal body length*; thus, broad-sense heritabilities are defined here as the proportion of genetic to expected phenotypic variance when body size is kept constant between individuals. Statistical significance for heritability of the traits or of their flexibility were assessed by comparing the full model to a model with no *clone* or period*clone random effect (likelihood ratio test, library *lme* [84]). Bootstrapping was used to compute mean values and confidence intervals for significant heritabilities (1000 resampling with replacement [86]).

*Clutch size* (for the analysis of egg size), *maternal body length* and *period* were treated as fixed effects. Statistical significance was assessed with log likelihood ratio tests [84] and model parameters were estimated by the restricted log-likelihood method. The *lme* package [84] was used to check the assumptions of models including mixed effects; variables were transformed whenever necessary. Robustness to outliers was tested by removing observations with large Cook distances; only robust results are presented here.

#### Phenotypic and genetic correlations

**Figure 8:**
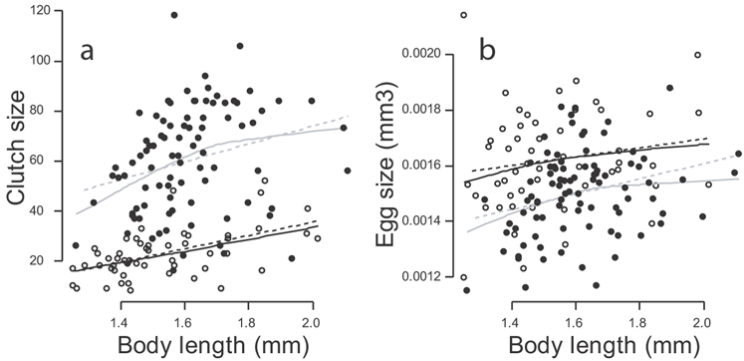
Clutch size, egg size and maternal body length. Clutch size (A) and egg size (B) as a function of maternal body length per period (open circles: P1; closed circles: P2). Egg size increases with maternal body length (χ^2^_1_ = 4.73, P = 0.029) similarly in both periods (χ^2^_1_ = 0.819, P = 0.36). These relations are materialized by regression lines (dotted lines) and lowess nonparametric regression lines (continuous line) for both P1 (black) and P2 (gray).

Correlations between egg size and clutch size were analysed by modelling *egg size* and *clutch size* with two independent linear mixed models, using *maternal body length* and *period* as fixed effects, and an interaction between *clone* and *period* as a random effect. Both traits were dependent additively on *maternal body length* and *period* (Figure 8). For each period, phenotypic correlations between egg size and clutch size were studied by correcting these variables for maternal body length (they were scaled to 1.6 mm, mean female length during the experiment, see Figure 3a). Similarly, the variables plotted and analyzed in Figure 1 and Figure 4 are controlled for maternal body length and scaled to a 1.6 mm long female. Within-clone phenotypic correlations were computed using model residuals, thus controlling for both maternal body length and genetic variation (Figure 3b). For each period, genetic correlations were sought between the genetic values of the traits, computed as the sum of the residuals of the models' random parts with the predicted value of the dependent variable for a 1.6 mm female. Genetic values of the flexibility of reproductive investment were computed as the difference between the genetic values of reproductive investment in each period.

#### Survival analysis

In the offspring survival experiment, mortality was analyzed with a Cox proportional hazards model (*Coxph* function from package survival, R 2.1 [87]). In order to fulfil the Cox proportional hazard assumptions, only the first 80% death events were included in the analysis for each food treatment; this threshold was reached within 50 days for the no-food treatment, and within 115 days for the *ad libitum* food treatment (Figure 2b). The potential for mortality correlation among groups of sisters within clutches was taken into account by computing a robust variance (*cluster* option). Ration was treated as a stratum variable (*strata* option) to allow for non proportional hazards when comparing the effect of egg size between the two food treatments [88]. The effect of egg size on survival is illustrated in Figure 2b & 2c by means of a graphical method that consists of plotting measurements of egg size against the residuals of a Cox model (known as martingale residuals [89]) that does not include egg size as a covariate. Martingale residuals can be interpreted as an excess of death given the model: positive values mean that the corresponding data have a shorter lifespan than predicted by the model whereas measurements with negative residuals have a longer lifespan than predicted. Therefore plotting these residuals against egg size reveals the underlying relationship between this variable (egg size) and the hazard rate (mortality).

#### Cluster analysis

To build-up a life history distance tree (Figure 6), we used a hierarchical cluster analysis (*hclust* function in program R 2.1, single linkage method) performed on the genetic values (centred and standardized) of egg size and reproductive investment expressed in the second period (cf. Figure 4c).
